# Pragmatic Competence Modulates Counterfactual Emotion Processing: Eye-Tracking Evidence from Mandarin Chinese

**DOI:** 10.3390/bs16071164

**Published:** 2026-07-10

**Authors:** Haoyun Dai, Yanhao Xu, Jing Bian

**Affiliations:** School of Foreign Languages and Cultures, Nanjing Normal University, Nanjing 210023, China; 04448@njnu.edu.cn (H.D.);

**Keywords:** counterfactual emotions, pragmatic competence, eye-tracking, regret, relief, narrative comprehension

## Abstract

Counterfactual thoughts about “what might have been” typically evoke emotions such as regret and relief, yet little is known about how such emotions are processed during real-time language comprehension. This eye-tracking study examined how native Chinese readers infer counterfactual emotions in narratives and whether pragmatic competence modulates this process. Participants read stories in which a character made a decision and subsequently experienced a positive or negative outcome; the final sentence was either consistent or inconsistent with the character’s feeling of regret or relief. Emotion words that mismatched the counterfactual context elicited longer reading times than matching words, indicating rapid online inference and monitoring of the protagonist’s emotions. Crucially, this emotional-consistency effect emerged earlier for relief than for regret (in first-pass reading times for relief but only in later eye-movement measures for regret), revealing an early processing advantage for relief. Moreover, individual differences in pragmatic competence selectively modulated inconsistency detection for regret but not relief: higher pragmatic competence was associated with greater early sensitivity to regret-related mismatches. These findings suggest that inferring regret places greater demands on higher-order pragmatic processing than relief and point to an optimism bias in counterfactual thinking among Chinese readers.

## 1. Introduction

People spontaneously generate counterfactual alternatives to reality when considering how the past could have turned out differently if only they had made a different choice (e.g., If Julia had taken the bus as usual instead of walking to work, she wouldn’t have gotten wet.). Such counterfactual thoughts serve important cognitive and affective functions, such as drawing causal inferences, adjusting future behavior and modulating emotional experience (Byrne, 2016). Yet communicating counterfactual thoughts through language can be pragmatically challenging because such expressions convey information that is factually false, requiring listeners to move beyond the literal meaning to infer the speaker’s intentions and emotional states (Kulakova & Nieuwland, 2016; Dai et al., 2019). Previous cognitive studies have primarily focused on the semantic-pragmatic aspects of counterfactual processing, examining how people use contextual information to compute the dual meaning of counterfactuals, namely, the literal supposition and the implied reality (e.g., Ferguson, 2012; Ferguson & Cane, 2015; Dai et al., 2021). However, limited attention has been given to the affective-pragmatic aspect of counterfactual processing, which arises from the contrastive appraisal of reality against counterfactual alternatives. The present study addresses this gap by investigating how people infer and track others’ emotional states in Chinese counterfactual contexts, and testing whether individual differences in pragmatic ability impact the online processing of counterfactual emotions.

### 1.1. Counterfactuals and Pragmatic Inference

Human communication is effective because people generally adhere to the conversational maxims based on the cooperative principle (Grice, 1975). Central to this is the Maxim of Quality, which stipulates that speakers should not say what they believe to be false or lack evidence for. Counterfactual statements, however, constitute a special case, as they deliberately convey information that is contrary to fact. Successful counterfactual comprehension therefore relies on pragmatic inference to move beyond literal falsity, reconstruct the implied reality, and infer the speaker’s intentions.

This process of pragmatic inference has been evidenced in a number of empirical studies (e.g., Ferguson, 2012; Ferguson & Cane, 2015; Ferguson et al., 2019; Kulakova et al., 2013, 2014; Kulakova & Nieuwland, 2016). For instance, in an EEG study on German conditionals, Kulakova et al. (2014) reported a late transient negativity for subjunctive auxiliaries (which mark counterfactuality) relative to indicative auxiliaries during sentence comprehension. This effect was interpreted as reflecting increased inferential processing demands associated with counterfactuals’ dual representation and showed that comprehenders are already sensitive to the pragmatic implications of counterfactuality at the earliest point where it is marked by subjunctive mood. Subsequent study (Kulakova & Nieuwland, 2016) further revealed that words consistent with real-world knowledge elicited greater semantic processing costs (enlarged N400 effects) in counterfactual conditionals than in indicative conditionals (e.g., If sweets were/are made of *sugar*…). This suggested that upon encountering the subjunctive mood, comprehenders pragmatically inferred that information presented in a counterfactual context should not be taken as true, thereby reducing their reliance on factual knowledge in subsequent semantic processing. Notably, the magnitude of this pragmatic-counterfactual N400 effect correlated positively with individuals’ pragmatic ability, underscoring the role of pragmatic competence in enhancing sensitivity to the falsity of counterfactual premises. Converging evidence for the active involvement of pragmatic inference in counterfactual processing also comes from neuroimaging studies. Relative to factual or indicative sentences, counterfactuals elicit increased activation in right inferior frontal and temporo-parietal regions that are commonly implicated in non-literal meaning construction, contextual integration, and mentalizing (e.g., Nieuwland, 2012; Kulakova et al., 2013; T. Y. Wang & Xu, 2025).

In contrast to Indo-European languages, which have morphological/syntactic markers to signal entry into the counterfactual world, Chinese lacks overt grammatical devices dedicated to encoding counterfactuality. As a result, counterfactual interpretations in Chinese are typically derived from the interaction of multiple counterfactuality-enhancing cues, such as temporal reference, negation, encyclopedic knowledge, and contextual information (e.g., Jiang, 2011, 2019; C. H. Wang, 2016; Yong, 2015, 2016). Corpus-based analyses revealed that among these cues, contextual information constitutes the most frequent and effective source for deriving counterfactual interpretations (C. H. Wang, 2016; Yong, 2016). Pragmatic inference grounded in contextual information is therefore particularly critical for counterfactual comprehension in Chinese. This typological characteristic has motivated a growing body of experimental work on Chinese counterfactual processing (e.g., Dai et al., 2019, 2021; Dai & Ni, 2023; T. Y. Wang & Xu, 2022; Liao et al., 2023; Kong et al., 2024). For instance, an ERP study (Dai et al., 2021) showed that the time course of anomaly detection in Chinese counterfactuals is strongly modulated by the transparency of contextual cues, with pragmatically inferred counterfactuals being processed more efficiently than those based on explicit violations of encyclopedic knowledge. More recent studies further demonstrate that linguistic-pragmatic cues such as personal perspective (T. Y. Wang & Xu, 2022), as well as negation and temporal indicators (Liao et al., 2023; Kong et al., 2024) play an important role in Chinese counterfactual processing.

However, from a broadly pragmatic view of utterance interpretation, counterfactual comprehension is expected to be jointly shaped by properties of the linguistic input and characteristics of the comprehender. Yet this line of research has primarily focused on language-internal factors (e.g., structural and semantic properties of counterfactuals), and empirical evidence on how individual differences in pragmatic ability shape the processing of Chinese counterfactuals remains scarce. Given the evidence reviewed above indicating that counterfactual meaning construction, particularly in Chinese, crucially relies on pragmatic inferencing, individual differences in social communicative abilities and pragmatic skills are therefore expected to play a pivotal role in modulating Chinese counterfactual processing. Addressing this issue is one of the key objectives of the present study.

### 1.2. The Emotional Processing of Counterfactuals

Extant studies on counterfactual processing, as in much pragmatic research, have primarily focused on propositional meaning while overlooking the emotional dimensions of language use. Recent years have witnessed the rise of affective pragmatics, which proposes that pragmatics should move beyond propositional meaning to account for affective effects, highlighting emotions as central drivers of speech acts and pragmatic inference (e.g., Wharton & de Saussure, 2024; Scarantino, 2017; Hoemann et al., 2025; Zhuo, 2024). In this respect, counterfactuals provide an ideal lens for examining the interplay between emotion and pragmatic inference. From the functionalist perspective (e.g., Roese, 1994, 1997), counterfactuals serve two primary psychological functions: an affective function that regulates emotions via upward or downward contrasts between imagined alternatives and actual reality; and a cognitive function that supports causal inference and planning for future actions. Comparative and corpus studies revealed that English counterfactuals are commonly used for both emotional expression and causal reasoning, whereas Chinese counterfactuals are predominantly employed as a means of emotional catharsis when narrating personal experiences and seldom function as tools for logical inference or analytical reasoning (e.g., Wu, 1994; Yuan, 2015; Jia, 2019). Therefore, emotion processing constitutes a central driving force for understanding Chinese counterfactuals and their pragmatic implications.

Counterfactual emotions can be positive or negative, depending on the direction of contrastive appraisal between a hypothetical alternative and the actual reality. When the hypothetical alternative is better than reality (known as an upward counterfactual; Roese, 1997; Epstude & Roese, 2008), negative feelings of regret will be elicited. Conversely, when the hypothetical alternative is worse than reality (known as a downward counterfactual), positive feelings of relief are typically induced. Developmental studies showed that counterfactual emotions emerge later than basic counterfactual thinking (e.g., McCormack et al., 2016; Weisberg & Beck, 2012)—typically around the age of 5 years old—and that the understanding and experience of regret develop earlier than relief (e.g., Guttentag & Ferrell, 2004, 2008). Children with autism exhibited specific deficits in identifying and explaining relief in downward counterfactuals, but not regret in upward counterfactuals (e.g., Begeer et al., 2014). Studies with adults also revealed that counterfactuals were more commonly generated following a bad outcome, and negative feelings of regret were more frequently experienced in counterfactual contexts (e.g., Roese, 1997; McEleney & Byrne, 2006; Weisberg & Beck, 2010). Taken together, these findings suggest that the processing of counterfactual emotions is direction-sensitive, with upward and downward comparisons relying on partially distinct cognitive mechanisms. Yet, how these mechanisms operate in real time during language comprehension remains largely unexplored.

So far, most studies in this area have adopted production paradigms, primarily examining how people generate counterfactual alternatives. Much less attention has been given to how people infer and track others’ counterfactual emotions as language comprehension unfolds. In a relevant study, Black et al. (2019) employed an eye-tracked reading task to examine the real-time inference of counterfactual emotions during language comprehension. The participants were presented with counterfactual narratives in which a character made a decision and subsequently experienced either a positive or negative outcome. The final sentence of each story contained a remark about the character’s mood that was either consistent or inconsistent with the character’s expected feeling of regret or relief. Results revealed that typically developing (TD) adults showed the same time course of sensitivity to inconsistencies in the regret and relief conditions, suggesting that the pragmatic inference of counterfactual emotions in upward and downward directions reached comparable levels of processing. This pattern seems to contrast with findings from developmental and production studies. One possible explanation concerns the task modality: eye-tracking measures can better capture implicit cognitive processes during comprehension, but previous explicit, response-based measures may primarily reflect the expressive dimension of counterfactual thinking. Another possible explanation concerns individual differences in counterfactual emotion processing. Unlike simple emotional responses such as disappointment or contentment, which arise directly from the polarity of an outcome, regret and relief are second-order emotions that involve recursive inference or reflection on one’s prior intentions and decisions (e.g., Begeer et al., 2014). Therefore, counterfactual emotion processing is likely to be influenced by individual differences in pragmatic and inferential abilities. In Black et al.’s (2019) study, adults with ASD, a pervasive developmental disorder characterized by social difficulties and pragmatic impairments, exhibited distinct processing patterns from TD adults in the relief condition but not in the regret condition. This suggested that communicative and pragmatic ability may modulate emotion processing differently for upward versus downward counterfactuals. Further research is needed to elucidate whether and how such modulation operates in typically developing populations.

Extant studies on the emotion processing of Chinese counterfactuals have been very limited, despite their inherently affective nature. However, existing findings provide some evidence that the emotion patterns embedded in Chinese counterfactuals differ from those observed in Indo-European languages. For instance, Yuan (2015) conducted a corpus analysis of both ancient and contemporary Chinese, and extracted 122 counterfactual sentences from corpora, among which 101 were used to express emotions. Notably, of these 101 instances, 72 conveyed feelings of relief, whereas only 29 expressed regret. This asymmetrical distribution was consistent across both ancient and contemporary corpora. The same pattern was further supported by a subsequent production study (Yuan & Zhang, 2016), in which researchers conducted semi-structured interviews with Chinese undergraduates and collected 114 counterfactual sentences from their natural conversations. The interview questions are designed to guide the participants to reflect on the big events that had made a difference in their lives and work. The results likewise revealed an overall optimistic tendency in Chinese counterfactual thinking: the participants more frequently generated “downward” counterfactuals (i.e., imagining how a situation would have turned worse if they had not made the previous decision) to express relief or contentment with their current circumstances. These results contrast with findings from Indo-European languages, where speakers tend to generate more “upward” counterfactuals reflecting on how a negative outcome could have been avoided and to express feelings of regret. However, the current evidence for Chinese counterfactual emotions has been derived primarily from production paradigms. Whether this cultural difference extends to real-time language comprehension remains to be determined. Building on the above observations, the influence of pragmatic ability on emotion inference may also manifest differently in Chinese counterfactuals. Addressing these issues is essential for clarifying how linguistic typology and pragmatic competence jointly shape the cognitive and emotional mechanisms that underlie counterfactual processing, thereby refining our understanding of the interface between affect and pragmatic inference in real-time meaning construction.

### 1.3. The Present Study

The present study sought to examine how native Chinese speakers make counterfactual inferences about others’ emotions during real-time language processing, and whether this process is modulated by individual differences in pragmatic competence. Following Black et al. (2019), we used an eye-tracking reading paradigm in which participants read counterfactual narratives that described negative or positive outcomes of a character’s explicit decision (e.g., Xiao Zhu got wet in the rain this morning because she chose to walk to work). Each narrative context was designed to prompt readers to consider how things could have been different, that is, whether a better or worse outcome than reality might have occurred if the character had made a different choice, and to elicit feelings of regret or relief for the character. For example, getting drenched because she chose to walk to work should make Xiao Zhu feel regretful, as reflecting on this negative outcome evokes counterfactual thoughts about how things could have been better (e.g., if she had taken the bus, she would have stayed dry). In contrast, avoiding getting drenched because she took the bus should make her feel relieved, as this positive outcome may evoke thoughts about how things could have been worse (e.g., if she had walked, she would have gotten wet). The final sentence of each narrative provided an explicit statement of the character’s mood after engaging in counterfactual reflection about the decision (e.g., She felt *upset/glad* about her choice). The critical word was either typically associated with emotional state of regret (e.g., frustrated, upset, bad) or relief (e.g., glad, happy, good). We compared conditions in which the emotion word was consistent or inconsistent with the character’s expected mood, so as to determine how quickly readers detect the incongruity, and how they recover from such difficulty during real-time inference of counterfactual emotions.

We expected that inconsistent emotion words would elicit longer reading times than consistent words, reflecting emotion inference based on counterfactual context. Importantly, this inconsistency effect may exhibit different processing patterns depending on the direction of counterfactual comparisons (upward vs. downward), which correspond to regret and relief, respectively. Regarding how the processing patterns may differ between regret and relief inference, we proposed two competing predictions grounded in prior research. First, based on the developmental trajectory of counterfactual emotion understanding and extensive evidence from production studies (e.g., Guttentag & Ferrell, 2008; Roese, 1997; Weisberg & Beck, 2010), we expected a stronger or earlier inconsistency effect during the inference of regret than relief. Specifically, inconsistent emotion words might disrupt reading immediately in the regret context (e.g., reflected in first-pass reading times on the critical word), whereas the disruption in the relief context may emerge later (e.g., in total reading times or post-critical regions). Alternatively, based on emerging evidence from Mandarin corpus and production studies (e.g., Yuan, 2015; Yuan & Zhang, 2016), counterfactual emotion processing in Chinese may exhibit a different pattern from that observed in Indo-European languages. In particular, due to an optimistic bias in counterfactual thinking among Mandarin speakers, readers may be more inclined to generate downward counterfactuals that emphasize satisfaction rather than regret. Under this account, the processing mechanism may show the reverse pattern, namely, a faster or stronger inconsistency effect in the relief condition than in the regret condition. Testing these predictions would allow us to examine whether Chinese counterfactual emotion processing follows general developmental patterns or reflects culture-specific cognitive tendencies.

Regarding the role of pragmatic competence in modulating counterfactual emotion processing, we predicted that the inconsistency effect would be stronger or earlier in individuals with higher pragmatic competence. This prediction rests on the grounds that inferring a character’s emotional state from a contrastive evaluation between the outcome and the character’s prior decision requires active engagement of pragmatic reasoning, which is more efficiently supported in individuals with stronger pragmatic skills. We further predicted that this modulation might differ between regret and relief conditions. Specifically, the modulation should be more pronounced in the emotion condition that imposes greater cognitive demands for native Chinese speakers, depending on which condition, as addressed in our first research question, elicits more effortful counterfactual reasoning.

## 2. Methods

### 2.1. Participants

Fifty native Chinese speakers from Nanjing Normal University took part in the present study. All received compensation for their participation. Four datasets were excluded from the analysis due to calibration problems, leaving data from 46 participants for formal analysis (32 women; mean age, 20.8 years; range, 18–27 years). All participants had normal or corrected-to-normal vision and reported no diagnosed reading difficulties. This study was carried out in accordance with the Declaration of Helsinki and was approved by the Ethics Committee of Nanjing Normal University.

### 2.2. Materials

Thirty-two experimental items were constructed and modified from Black et al. (2019), as shown in Table 1. Each experimental item was created in four versions corresponding to the four experimental conditions. Using a Latin-square design, each participant read only one version of each item. Each item consisted of 6 to 8 sentences and described a story in which a character made a decision that led to either a positive or negative outcome, thereby eliciting feelings of regret or relief. Passages began by introducing the context and the character, then two alternative choices were specified. Each scenario was designed so that the outcome was a “near miss”. For instance, the character chose to do something different from what they usually do, and they “only just” obtained or missed out on something. To better engage readers in counterfactual thinking, we modified the “because” structure in the final sentence of the original version (Because [character] did X, instead of Y, they felt…) into a “if” structure: “If [character] did Y, instead of X, [alternative consequence…]. They felt…”. Another consideration for changing the structure is that previous studies have suggested that positively valenced scenarios may inherently process faster than negatively valenced ones (Black et al., 2019; Lüdtke & Jacobs, 2015), which may confound the results of counterfactual emotion processing. The counterfactual framing in the revised version can introduce an oppositional scenario, thereby helping to balance the valence across conditions.

The critical word of experimental stimuli is the emotion word in the final sentence “He/She felt…about the decision he/she had made”. In consistent conditions, the valence of the word matched the polarity of the decision outcome. In inconsistent conditions, they were mismatched. This resulted in a within-subject design that crossed emotion (relief vs. regret) and consistency (consistent vs. inconsistent). Critical words were matched across conditions for word length, number of strokes, and word frequency (*ps* > 0.1). Experimental items further underwent three pre-tests for critical word plausibility, cloze probability, word valence and arousal.

Critical word plausibility was tested by 33 students from Nanjing Normal University. Participants were asked to rate how plausible the word was in the story context on a 7-point Likert scale (1 = highly implausible, 7 = highly plausible). Results showed that consistent critical words (*M* = 6.37) were rated significantly more plausible than inconsistent words (*M* = 1.41), *F*(1,31) = 22,303.75, *p* < 0.001. Emotion types, or the Emotion × Consistency interaction, did not influence ratings [*F*(1,31) = 1.225, *p* = 0.277; *F*(1,31) = 2.458, *p* = 0.120], suggesting that our stimuli were matched in terms of critical word plausibility across relief and regret conditions.

Cloze probability was assessed by a different set of 36 participants. Passages were truncated before the critical word, and participants were instructed to complete the sentence with the first sensible word that came to mind. Cloze probability was calculated as the percentage of trials that elicited the intended critical word. Results showed that participants were significantly more likely to complete sentences with consistent critical words (*M* = 62%) than inconsistent critical words (*M* = 1%), *F*(1,31) = 350.958, *p* < 0.001, and the Emotion (relief vs. regret) did not modulate this difference (*F*s < 1.5, *p*s > 0.1).

Finally, we invited another group of 30 students to rate the valence and arousal of the critical words on a 7-point Likert scale. Positive words had significantly higher valence ratings (*M* = 5.65) than negative words (*M* = 2.47), *t*(31) = 47.971, *p* < 0.001, but arousal ratings did not differ, *t*(31) = −0.310, *p* = 0.759.

Four presentation lists were created using a Latin square design, such that each participant read only one version of each experimental item. Each list contained 32 experimental items (8 per condition). To prevent participants from placing undue emphasis on experimental manipulation or developing task-specific processing strategies, 32 filler passages were constructed and randomly interspersed among experimental items. Equal numbers of participants were randomly assigned to one of the four trial lists.

### 2.3. Procedure

Testing took place in a sound attenuated, dimly lit lab room. Participants’ dominant eye was tracked using an EyeLink 1000 Plus eye-tracker (SR Research Ltd., Ottawa, Canada). Head movement was minimized with a fixed chin rest. Passages were presented in size 18 Song style on a VDU about 70 cm from the participants’ eyes. Prior to the formal experiment, the procedure was explained and participants were instructed to read naturally at their normal rate. Calibration was performed using a nine-point procedure. Before each trial, participants performed a drift correction using a central-fixation point. They then fixated on a marker at the top left of the screen where the first character of the upcoming passage would be displayed. Passages were displayed on the screen until participants finished reading and pressed the space bar to move on. For the purpose of keeping participants focused and attentive, each trial was followed by a yes/no comprehension question that probed the understanding of the plots in the story (e.g., Xiao Zhu went to work by bus today. Yes < > No).

The testing began with four practice trials to ensure participants understood the task. Following that, experimental stimuli and fillers were presented to participants in four blocks, with 16 trials each. Participants took a 3-min break between blocks. After the eye-tracking experiment, participants completed the Communication subscale of the Autism-Spectrum Quotient (AQ; Baron-Cohen et al., 2001). Although originally designed for assessing autistic traits, the AQ-Communication subscale has demonstrated good reliability and structural validity in neurotypical adult samples and can effectively capture individual differences in pragmatic ability (Broadbent et al., 2013). It has therefore been widely used in studies of pragmatic language processing in typical adults (e.g., Kulakova & Nieuwland, 2016; Spotorno & Noveck, 2014). This scale consists of 10 items assessing individuals’ communicative ability and language use in a social context (e.g., “I find it easy to ‘read between the lines’ when someone is talking to me”, or “Other people frequently tell me that what I’ve said is impolite, even though I think it is polite”), with a focus on conversational reciprocity and the ability to understand nonliteral language. The score of this scale ranges from 0 to 10, with higher scores indicating stronger presence of pragmatic deficits. The entire experiment took about 1 h to complete.

## 3. Results

### 3.1. Methods of Analysis

The experimental passages were divided into three regions for analysis (see Table 1): pre-critical region (two words preceding the critical word, e.g., 她很 “she felt very”), critical region (the emotion word in the last sentence, e.g., 庆幸 “glad”), and post-critical region (two words following the critical word, e.g., 自己). An automatic procedure pooled fixations shorter than 80 ms with larger adjacent fixations, excluded fixations shorter than 40 ms that were not within three characters of another fixation, and truncated fixations longer than 1200 ms (e.g., Black et al., 2019; Dai & Ni, 2023).

Three early measures of language processing were reported. *First fixation duration* refers to the duration of the initial fixation in a region and is associated with initial lexical processing/access. *First-pass reading time* (also known as gaze duration) sums all fixations in a region from first entering it until first leaving it and is usually taken to indicate the costs of early text processing. *Regression path reading time* is the sum of fixations from first entering a region on the left, to first exiting the region on the right, and it includes any regressive fixations back into earlier portions of text. This measure provides an indicator of integration difficulty and reanalysis process. We also analyzed one later measure. *Total reading time* is the sum duration of all fixations made within a region and provides an indication of the overall amount of time spent processing text in that region. The mean values for each of these four measures are shown in Table 2 for each region and each condition.

Eye movement data was log-transformed prior to analysis to increase normality. Linear mixed-effects models were constructed using the lmer function in the lme4 package (Bates et al., 2015) in R (version 4.0.2, R Core Team, 2020). Separate models were constructed for each region and measure. Each model included fixed effects of Emotion and Consistency. The two levels of each fixed effect were deviation coded (−0.5 vs. 0.5) to enable direct comparison. The maximal random effects structure was used, including random effects for participants and items, crossed random slopes of Emotion and Consistency within items, and crossed random slopes of Emotion and Consistency within participants (as suggested by Barr et al., 2013). When random effects led to non-convergence due to overparameterization, we removed them from the models. Statistical effects from these models are summarized in Table 3.

**Table 2:** Mean (SD) values for each reading measure, sentence region, and condition.

	Regret		Relief	
	Consistent	Inconsistent	Consistent	Inconsistent
*Pre-critical region*				
First fixation duration (ms)	198 (78)	195 (59)	213 (87)	206 (69)
First-pass reading time (ms)	205 (86)	199 (71)	229 (104)	221 (100)
Regression path reading time (ms)	214 (94)	209 (87)	243 (131)	242 (132)
Total reading time (ms)	249 (171)	278 (207)	256 (148)	287 (184)
*Critical region*				
First fixation duration (ms)	222 (90)	227 (91)	216 (84)	237 (100)
First-pass reading time (ms)	235 (110)	237 (100)	226 (97)	259 (117)
Regression path reading time (ms)	305 (181)	346 (222)	309 (197)	370 (230)
Total reading time (ms)	296 (170)	366 (235)	286 (157)	378 (231)
*Post-critical region*				
First fixation duration (ms)	211 (84)	210 (83)	212 (98)	200 (73)
First-pass reading time (ms)	220 (115)	216 (90)	215 (101)	207 (83)
Regression path reading time (ms)	417 (281)	550 (372)	422 (299)	582 (429)
Total reading time (ms)	279 (174)	308 (190)	271 (160)	319 (199)

**Table 3:** Model estimate, Standard Error (SE) and t-value for eye-tracking measures across regions, where * *p* < 0.05, ** *p* < 0.01, *** *p* < 0.001.

	First Fixation Duration			First-Pass Reading Time			Regression Path Time			Total Reading Time		
	*Est.*	*SE*	*t*-Value	*Est.*	*SE*	*t*-Value	*Est.*	*SE*	*t*-Value	*Est.*	*SE*	*t*-Value
*Pre-critical region*												
Emotion	0.023	0.012	1.929	0.039	0.013	2.907 **	0.044	0.015	2.964 **	0.016	0.018	0.864
Consistency	−0.002	0.012	−0.159	−0.006	0.013	−0.436	−0.001	0.015	−0.088	0.035	0.018	1.954
Emotion: Consistency	−0.019	0.025	−0.771	−0.016	0.027	−0.586	0.001	0.03	0.037	−0.004	0.036	−0.111
*Critical region*												
Emotion	0.002	0.009	0.191	0.008	0.010	0.809	0.016	0.013	1.189	0.005	0.013	0.35
Consistency	0.023	0.009	2.519 *	0.032	0.010	3.140 **	0.065	0.014	4.779 ***	0.092	0.013	6.968 ***
Emotion: Consistency	0.023	0.019	1.217	0.045	0.020	2.251 *	0.035	0.027	1.307	0.034	0.026	1.283
*Post-critical region*												
Emotion	−0.014	0.01	−1.372	−0.015	0.011	−1.405	0.017	0.018	0.924	0.002	0.015	0.148
Consistency	−0.007	0.01	−0.697	−0.005	0.011	−0.507	0.121	0.018	6.602 ***	0.050	0.015	3.250 **
Emotion: Consistency	−0.015	0.02	−0.755	−0.010	0.021	−0.447	0.030	0.036	0.834	0.024	0.030	0.800

### 3.2. Eye-Movement Results

#### 3.2.1. Pre-Critical Region

There was a significant effect of Emotion in first-pass reading times and regression path reading times, showing that participants spent longer reading times when the preceding narrative depicted a relief context than when it depicted a regret context (first-pass: *M* = 225 vs. 202, regression path: *M* = 242 vs. 211). This result is similar to Black et al. (2019), who found that participants were more likely to regress back to re-read previous text following a relief context compared to a regret context. Furthermore, we also found a marginally significant effect of Consistency in total reading times (*p* = 0.051), with participants spending longer overall reading the pre-critical region when the passage contained an inconsistent word (*M* = 282) compared to a consistent word (*M* = 252).

#### 3.2.2. Critical Region

**Figure 1:**
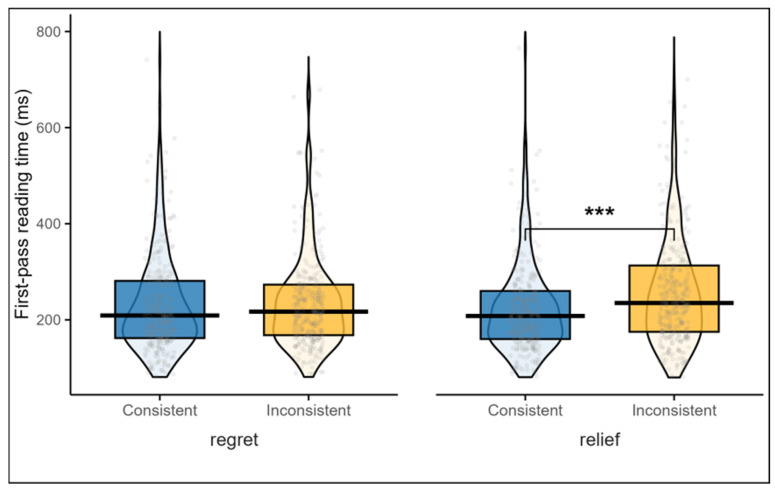
First-pass reading times (ms) on critical word across conditions. Asterisks indicate significant differences between consistent and inconsistent conditions (*** *p* < 0.001).

**Figure 2:**
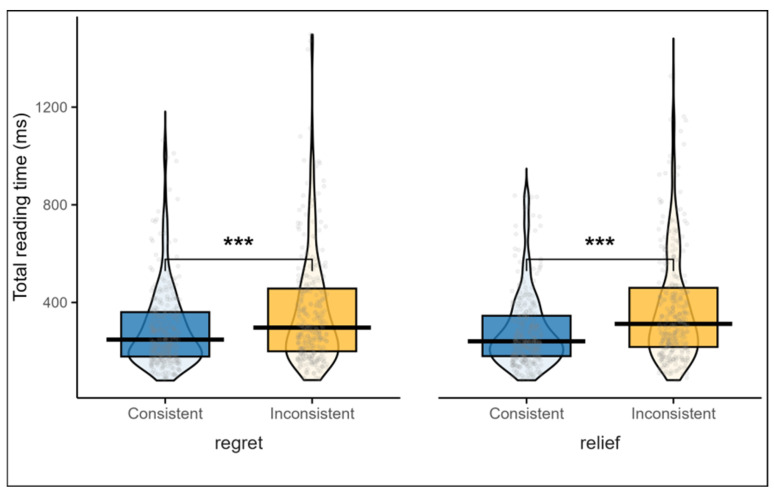
Total reading times (ms) on critical word across conditions. Asterisks indicate significant differences between consistent and inconsistent conditions (*** *p* < 0.001).

A significant main effect of Consistency was found across all four reading measures, with shorter reading times for consistent words than inconsistent words (first fixation: *M* = 219 vs. 232, first-pass: *M* = 230 vs. 248, regression path: *M*= 307 vs. 359, total reading: *M* = 291 vs. 372). Importantly, there was a significant Emotion × Consistency interaction in first-pass reading times. Follow-up analysis examined the consistency effect for regret and relief conditions separately. Results revealed that consistent word was processed faster than inconsistent word in the relief condition, *Est.*= −0.054, *SE* = 0.014, *t*= −3.835, *p* < 0.001, but no difference was found between the consistent word and the inconsistent word in the regret condition, *Est.*= −0.009, *SE* = 0.014, *t* = −0.624, *p* = 0.533. This Emotion × Consistency interaction was no longer significant in later reading measures of regression path reading time and total reading time, *ps* > 0.1. These results suggested that, in the early stage of emotion processing, participants immediately detected the inconsistency with the character’s expected feeling of relief, but were delayed in detecting regret (see Figure 1). In the later processing stage, participants eventually made appropriate inference about the character’s mood in both relief and regret conditions, and were sensitive to the critical word’s fit with the character’s counterfactual emotions (see Figure 2).

#### 3.2.3. Post-Critical Region

There was a main effect of Consistency in regression path reading time and total reading time, with longer reading times following an inconsistent critical word than a consistent word (regression path: *M* = 565 vs. 420, total reading: *M* = 313 vs. 275). No other effects reached significance. This spill-over effect of inconsistency shows that, by the post-critical region, participants remained sensitive to the congruence with the character’s emotions and had difficulty integrating subsequent information, and this later processing pattern did not differ between relief and regret conditions.

### 3.3. Correlations with AQ-Communication Scores

**Figure 3:**
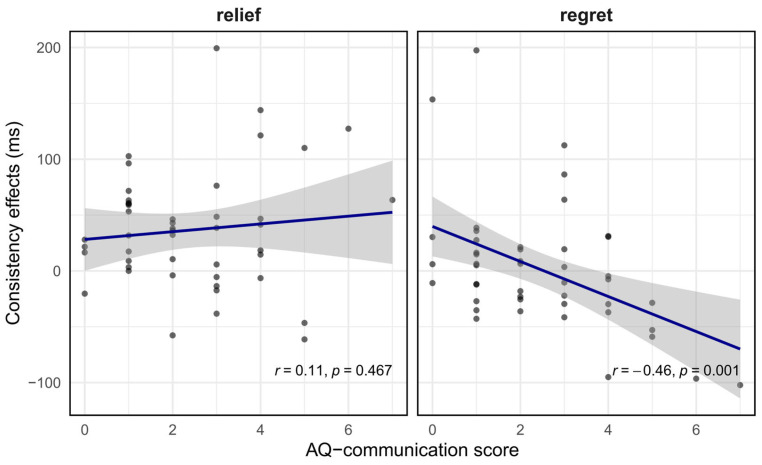
Correlation of the AQ-Communication score with the consistency effect (inconsistent conditions minus consistent conditions) in first-pass reading times, examined separately for the relief and regret contexts.

For this part, we examined whether and how individuals’ pragmatic skill modulates emotion processing in counterfactual contexts. We first calculated the individual consistency effect (the reading time difference between inconsistent and consistent words) in relief and regret conditions separately. This constitutes an index of individual sensitivity to the pragmatic violation of counterfactual emotions, with larger positive values indicating stronger sensitivity. The correlation analyses were conducted between individuals’ AQ-Communication score and consistency effect for all four reading measures in the critical region. Results revealed that individual pragmatic proficiency was significantly correlated with the consistency effect in first-pass reading times under the regret condition, *r*(44) = −0.46, *p* = 0.001, with pragmatically skilled participants (low AQ-Communication score) showing larger positive consistency effects than participants with low pragmatic skills (high AQ-Communication score). But the correlation was not significant under the relief condition (see Figure 3), *r*(44) = 0.11, *p* = 0.467. No other correlations were obtained with later reading measures (i.e., regression path reading time and total reading time), *p*s > 0.1. This suggests that individual pragmatic competence modulates the processing of counterfactual emotions only in regret conditions, and only during the early processing stage.

## 4. Discussion

In this paper, we examined how native speakers of Mandarin Chinese infer counterfactual emotions during real-time language comprehension and whether this process is modulated by individual differences in pragmatic competence. Two main findings emerged. First, although readers generally showed longer reading times for emotion words that were inconsistent with the prior counterfactual context, the time course of this inconsistency effect differed for relief and regret: in the relief condition, the effect was already visible in first-pass reading times, whereas in the regret condition it emerged only in later measures (regression-path time and total reading time). This pattern points to earlier sensitivity to positive counterfactual emotions and is consistent with an optimistic bias in Chinese counterfactual thinking. Second, individual differences in pragmatic competence modulated counterfactual emotion processing, and this modulation was specific to regret, with higher pragmatic skill being associated with greater early sensitivity to regret. This pattern suggests that regret may function as a more cognitively demanding counterfactual emotion, and that its online processing relies more heavily on pragmatic inference than relief. Taken together, the present results corroborate affective pragmatic accounts (e.g., Scarantino, 2017; Wharton & de Saussure, 2024) and Affective Language Comprehension model (e.g., van Berkum, 2018, 2019) by showing that emotion inference shapes real-time language processing in pragmatically enriched contexts, and that this process can be modulated by both cultural biases and individual pragmatic abilities.

### 4.1. The Emotion Processing in Chinese Counterfactuals

In the current study, we observed a rapid effect of emotional consistency: emotion words that were inconsistent with the prior counterfactual context elicited longer reading times than consistent words in the critical region. This pattern indicates that readers rapidly generate inferences about the character’s emotions based on the preceding context during the incremental build-up of a discourse representation, and that they continuously evaluate the fit between the expected emotional state and the incoming word. This finding converges with previous work suggesting that emotion inference and monitoring are automatic, online processes that unfold during comprehension, rather than a global evaluation made only at the end of the text (e.g., Gernsbacher et al., 1998; Zhang et al., 2017).

Notably, the emotional-consistency effect observed here emerged earlier than the semantic-consistency effects reported in previous studies on counterfactuals. In an eye-tracking study of Chinese counterfactual discourse comprehension, Dai and Ni (2023) examined emotion-neutral critical words that were either consistent or inconsistent with a prior counterfactual context. They found that effects of semantic inconsistency emerged only in later eye-movement measures (e.g., total reading time) and argued that constructing and maintaining the dual meaning of counterfactuals (the literal supposition vs. the implied reality) is cognitively demanding, thereby delaying the online detection of semantic anomalies. Similar delays have also been reported in several eye-tracking studies on English counterfactuals (e.g., Ferguson, 2012). By contrast, in the current study, the main effect of emotional consistency already emerged in early measures of first-pass reading time (despite a significant Emotion × Consistency interaction that we elaborate on below). This pattern suggests that, whereas constructing a dual-meaning representation of counterfactuals is relatively slow and cognitively demanding, the subsequent contrastive appraisal between the imagined supposition and the actual outcome, and the resulting counterfactual emotions, are computed relatively rapidly by native Chinese speakers. This interpretation is in line with previous work arguing that Chinese counterfactuals primarily engage consequence comparison mechanism to serve affective functions (Yuan, 2015; T. Y. Wang & Xu, 2022). The present findings are also consistent with the affective primacy hypothesis (e.g., Zajonc, 1980), which posits a processing advantage for emotional information: emotional features may be pre-activated and prioritized over other sources of semantic information (Delaney-Busch & Kuperberg, 2013; Hinojosa et al., 2020).

Beyond this general emotional-consistency effect, the eye-movement data revealed distinct time courses for relief and regret. In the relief condition (downward counterfactual), emotional anomaly detection was already evident in early measures, with longer first-pass reading times on inconsistent than consistent emotion words. In the regret condition (upward counterfactual), by contrast, disruption emerged only in later measures, namely regression-path time and total reading time. Building on discourse comprehension models (Kintsch, 1988; Zwaan et al., 1995), Zhang et al. (2017) proposed a two-stage process underlying emotion comprehension in discourse: an initial stage of monitoring and prediction, followed by a later stage of evaluation and model updating. Within this framework, our results suggest that early emotion monitoring and prediction based on contextual information is more efficiently or more readily engaged in relief contexts, whereas later measures indicate that both regret and relief ultimately reach comparable levels in the integration/updating process.

The present findings allow us to evaluate the two competing predictions proposed in the Introduction. The first prediction was grounded in a developmental perspective (e.g., Guttentag & Ferrell, 2004; Roese, 1997; Weisberg & Beck, 2010), according to which regret should be processed more efficiently than relief because it is acquired earlier during development and therefore becomes more cognitively accessible during online processing. The second prediction was motivated by a language-use perspective, drawing on Mandarin corpus analyses and elicited production studies (e.g., Yuan, 2015; Yuan & Zhang, 2016), which indicate that downward counterfactuals expressing relief are used more frequently than upward counterfactuals expressing regret in Chinese. The present eye-tracking data lend stronger support to the second prediction: emotional inconsistency was detected earlier in relief than in regret contexts, suggesting that Chinese readers inferred relief more readily during online comprehension.

This pattern therefore suggests that, in adult language comprehension, accumulated language experience and culturally preferred patterns of counterfactual thinking may play a more prominent role in shaping online processing than developmental acquisition alone. This interpretation is consistent with usage-based accounts of language processing (Ellis, 2002; Gries & Ellis, 2015), which propose that frequent patterns of language use become increasingly entrenched through experience and consequently shape online language processing. So far, most developmental evidence comes from Indo-European languages, and the limited evidence available in Mandarin-speaking children suggests a broadly comparable developmental trajectory, generally pointing to an earlier or more robust acquisition of regret than relief (Tong et al., 2013). However, these developmental findings concern the emergence of counterfactual emotions during childhood, whereas the present study examined their online processing in adulthood. It is therefore plausible that developmental acquisition primarily constrains the initial establishment of counterfactual emotion representations, whereas accumulated language experience and culturally preferred patterns of counterfactual thinking increasingly shape their online processing in adulthood. The convergence between the present eye-tracking findings and previous Mandarin corpus and production studies further supports this interpretation, suggesting that habitual exposure to downward counterfactual expressions may facilitate the online inference of relief during language comprehension.

It might be argued that the earlier emotion-consistency effect in the relief condition simply reflects a general positivity advantage, whereby positively valenced information is processed faster than negatively valenced information (e.g., Lüdtke & Jacobs, 2015). However, this explanation is unlikely for two reasons. First, unlike studies manipulating the valence of a single outcome, the present experiment explicitly introduced counterfactual scenarios in which the hypothetical outcome stood in opposition to the actual outcome. Consequently, the target emotion could not be determined from the valence of either outcome alone. Instead, readers were required to integrate and compare the actual and the hypothetical outcomes and infer the appropriate emotion from the contrast between them. Successful processing therefore depended on counterfactual emotion inference rather than simple positivity or negativity. Second, the eye-movement data do not support the pattern predicted by a general positivity account. If the earlier emotion-consistency effect merely reflected facilitated processing of positive information, one would expect processing advantages throughout the relief condition, including the pre-critical region. Instead, readers exhibited significantly longer first-pass and regression-path reading times in the pre-critical region for relief than for regret contexts, indicating that they invested additional processing effort in constructing and evaluating the relief counterfactual contexts before encountering the critical emotion word. Such deeper upstream processing may heighten readers’ sensitivity to the expected relief state and, in turn, facilitate the rapid detection of emotional mismatches at the critical region.

### 4.2. Pragmatic Competence Modulates Counterfactual Emotion Processing

The present study also revealed a selective modulation of counterfactual emotion processing by pragmatic competence. Individual differences in pragmatic competence affected the early detection of emotional inconsistency only in the regret condition: higher pragmatic ability was associated with greater first-pass sensitivity to regret-related mismatches, whereas relief processing showed no reliable modulation by pragmatic skill. These findings suggest that regret and relief differ not only in their processing dynamics but also in the inferential resources required for successful comprehension. Specifically, regret counterfactuals appear to place greater demands on higher-order pragmatic inference, whereas relief may rely more on a comparatively direct contrastive appraisal between the actual outcome and a worse alternative. Such downward comparisons may be facilitated by highly routinized evaluative constructions in Chinese, leaving less room for individual differences in pragmatic ability to exert an influence.

This interpretation is consistent with the Theory of Affective Pragmatics (TAP; Scarantino, 2017; Zhuo, 2024; Wharton & de Saussure, 2022), which conceptualizes emotion expressions as affective speech acts that differ in the relative prominence of cognitive, affective, and volitional components. Functional accounts of counterfactual thinking provide a complementary perspective for how regret and relief differ along these dimensions (Roese, 1997; Markman & McMullen, 2003). Specifically, upward counterfactuals that evoke regret primarily serve preparatory functions by encouraging reflection on how future outcomes might be improved, whereas downward counterfactuals that evoke relief mainly serve affective functions by enhancing positive feelings through comparison with a worse alternative. Accordingly, regret counterfactuals can be characterized as affective speech acts that integrate affective evaluation with responsibility attribution and an implicit orientation toward future behavioral adjustment. Relief counterfactuals, in contrast, primarily express positive affect arising from the avoidance of a worse outcome and convey a favorable evaluation of the actual outcome, with comparatively less emphasis on responsibility attribution and future-oriented considerations. In light of the present findings, we suggest that this asymmetry in affective-pragmatic profile places greater demands on comprehenders’ inferential resources during discourse comprehension, thereby making online regret inference more susceptible to individual differences in pragmatic competence than relief inference.

To our knowledge, direct evidence concerning individual differences in the online processing of regret and relief in healthy adult language users is currently lacking. Nevertheless, converging empirical evidence from decision-making and affective neuroscience is broadly consistent with the present interpretation. Neuroimaging studies suggest that regret engages neural systems supporting outcome evaluation and responsibility attribution to a greater extent than relief, whereas relief is more closely associated with reward-related circuitry and the down-regulation of negative affect (Coricelli et al., 2005). This functional distinction has been further corroborated by a recent neuroimaging meta-analysis, which identified partially dissociable neural systems underlying regret and relief, and linked regret to networks implicated in valuation, cognitive control, and self-referential processing (Varma et al., 2023). Although these findings come from different research paradigms, they consistently indicate that regret recruits richer evaluative and inferential processes than relief. The present findings extend this literature by demonstrating that these functional differences are also reflected in online discourse comprehension, where individual differences in pragmatic competence selectively influence the online inference of regret but not relief.

Whereas the preceding discussion explains why regret places greater affective-pragmatic demands than relief, the Affective Language Comprehension (ALC) framework (van Berkum, 2018, 2019; Hinojosa et al., 2020) provides a process-level account of how these differences unfold during online language comprehension. According to ALC, emotional meaning can be constructed at multiple representational levels, from lexical items to situation models, and subsequently to higher-level representations of speaker/character stance and social intentions. Once established, the higher-level representations can rapidly feed back to influence ongoing lexical processing. Within this framework, the different temporal profiles of relief and regret observed in the present study may reflect the different representational demands associated with emotion inference. Relief can often be predicted on the basis of relatively straightforward contrastive appraisals at the level of the situation model—recognizing that “things could have been worse, but turned out better than a negative alternative”. This relatively automatic process allows inconsistency effects to emerge early in first-pass reading, leaving little opportunity for individual differences in pragmatic competence to influence online processing. By contrast, regret requires readers to move beyond the situation model and establish a higher-level representation of the character’s evaluative stance and intentions. This process will rely more heavily on later integrative stages, consistent with our finding that inconsistency detection in the regret condition was only robustly observed in regression-path times. Importantly, establishing such higher-level stance representations is precisely the kind of higher-order inference that draws on pragmatic skills and mentalizing. Our finding that pragmatic competence modulated early first-pass measures only for regret suggests that readers with higher pragmatic ability can rapidly establish stance-level representations, enabling top-down predictions of emotional meaning during initial lexical processing. In contrast, readers with lower pragmatic competence appear to rely on more incremental integration processes, with full interpretation of regret emerging only during later stages.

Taken together, by combining individual-differences measures with fine-grained online indices, the present study illustrates how macro-level theories of affective pragmatics (Scarantino, 2017; Zhuo, 2024) and Affective Language Comprehension (van Berkum, 2018, 2019) can be anchored in concrete processing dynamics. Our findings highlight that different affective speech acts impose different levels of pragmatic inferential loads, and that these loads determine whether individual differences in pragmatic competence will matter and when their influence will surface during comprehension. Specifically, the present result pattern suggests that higher-order pragmatic resources are particularly critical for the rapid construction and monitoring of cognitively demanding counterfactual emotions such as regret, enabling stance-based inferences and predictions to guide processing from early stages. This provides an empirical bridge between work on affective pragmatics, online language processing and individual differences, showing that counterfactual emotions offer a particularly sensitive test case for how emotional appraisal and pragmatic inference interact in real-time comprehension.

## 5. Conclusions

The present study used an eye-tracking experiment to show that Chinese native speakers process the counterfactual emotions of regret and relief differently during online language comprehension, revealing an overall early processing advantage in inferring relief in others. The selective modulation of regret, but not relief, by pragmatic competence suggests that the more extended time course of inconsistency detection for regret may be related to additional affective-pragmatic inferential load, such as responsibility attribution and norm-based evaluation. These findings highlight the facilitating role of pragmatic ability in supporting higher-order emotion inference in real-time language processing and provide empirical evidence linking macro-level theories of affective pragmatics (TAP) and Affective Language Comprehension (ALC) to concrete temporal signatures in online comprehension.

Several limitations of the present study point to productive directions for future work. First, our materials targeted written Mandarin narratives and two counterfactual emotions that differ in their affective-pragmatic profiles. The next step is to examine whether the same asymmetry between regret and relief, and the same selective role of pragmatic competence, can be observed across languages and in more interactive settings, such as dialog and natural conversation, using a broader range of behavioral, neurophysiological, and multimodal measures. Furthermore, due to the lack of well-validated multidimensional measures of pragmatic competence for non-clinical adult populations, we operationalized pragmatic competence as a single composite index and related it to eye-movement measures in a correlational design. Future studies could dissociate different facets of pragmatic skill (e.g., sensitivity to conversational implicature, comprehension of irony, mentalizing-based perspective taking) or experimentally manipulate pragmatic demands in discourse. Such work would help to further specify which components of pragmatics most strongly constrain the inference of counterfactual emotion in real time.

## Figures and Tables

**Table 1:** Example stimuli and approximate translations by condition (underlined words indicate critical regions, forward slashes denote regions of analysis).

Relief Consistent	无论是步行还是坐公交车，小朱都要花半小时去上班。因为她喜欢锻炼身体，所以她几乎每天都选择步行。可是今天，小朱十分疲惫，于是她决定乘公交车去上班。正当她在车上找到座位并准备坐下时，外面突然下起了倾盆大雨。如果小朱早上没有乘公交车，而是像往常一样步行上班，那么她就要淋雨了。/她很pre-critical/庆幸critical/自己post-critical/做了这样的决定。
	It takes half an hour for Xiao Zhu to get to work whether she walks or takes the bus. She always chooses to walk because she enjoys exercising. However, today Xiao Zhu felt very tired, so she decided to take the bus to work. Just as she found a seat on the bus and was about to sit down, it suddenly started pouring rain outside. If Xiao Zhu had walked to work as usual, instead of taking the bus, she would have gotten drenched. She felt very glad about the decision she had made.
Inconsistent	无论是步行还是坐公交车，小朱都要花半小时去上班。因为她喜欢锻炼身体，所以她几乎每天都选择步行。可是今天，小朱十分疲惫，于是她决定乘公交车去上班。正当她在车上找到座位并准备坐下时，外面突然下起了倾盆大雨。如果小朱早上没有乘公交车，而是像往常一样步行上班，那么她就要淋雨了。/她很pre-critical/后悔critical/自己post-critical/做了这样的决定。
	It takes half an hour for Xiao Zhu to get to work whether she walks or takes the bus. She always chooses to walk because she enjoys exercising. However, today Xiao Zhu felt very tired, so she decided to take the bus to work. Just as she found a seat on the bus and was about to sit down, it suddenly started pouring rain outside. If Xiao Zhu had walked to work as usual, instead of taking the bus, she would have gotten drenched. She felt very upset about the decision she had made.
Regret Consistent	无论是步行还是坐公交车，小朱都要花半小时去上班。为了方便，她几乎每天都选择乘公交车。可是今天，小朱想锻炼一下身体，于是她决定步行去上班。小朱走了还没一会儿，外面突然下起了倾盆大雨。当她赶到单位时，她的衣服全被雨打湿了。如果小朱早上没有选择步行，而是像往常一样乘公交车上班，那么她就不会淋雨了。/她很pre-critical/后悔critical/自己post-critical/做了这样的决定。
	It takes half an hour for Xiao Zhu to get to work whether she walks or takes the bus. For convenience, she always chooses to take the bus. However, today Xiao Zhu wanted to get some exercise, so she decided to walk to work. Not long after she set out, it suddenly started pouring rain. By the time she arrived at the office, her clothes were completely soaked. If Xiao Zhu had taken the bus as usual, instead of walking to work, she would not have gotten wet. She felt very upset about the decision she had made.
Inconsistent	无论是步行还是坐公交车，小朱都要花半小时去上班。为了方便，她几乎每天都选择乘公交车。可是今天，小朱想锻炼一下身体，于是她决定步行去上班。小朱走了还没一会儿，外面突然下起了倾盆大雨。当她赶到单位时，她的衣服全被雨打湿了。如果小朱早上没有选择步行，而是像往常一样乘公交车上班，那么她就不会淋雨了。/她很pre-critical/庆幸critical/自己post-critical/做了这样的决定。
	It takes half an hour for Xiao Zhu to get to work whether she walks or takes the bus. For convenience, she always chooses to take the bus. However, today Xiao Zhu wanted to get some exercise, so she decided to walk to work. Not long after she set out, it suddenly started pouring rain. By the time she arrived at the office, her clothes were completely soaked. If Xiao Zhu had taken the bus as usual, instead of walking to work, she would not have gotten wet. She felt very glad about the decision she had made.

## Data Availability

The data that support the findings of this study will be available in Mendeley Data at [https://data.mendeley.com/datasets/x9vk36x4dc/1] following an embargo ending on 1 September 2026 (accessed on 5 July 2026).
